# A comparative study of protein synthesis in *in vitro *systems: from the prokaryotic reconstituted to the eukaryotic extract-based

**DOI:** 10.1186/1472-6750-8-58

**Published:** 2008-07-29

**Authors:** Jason R Hillebrecht, Shaorong Chong

**Affiliations:** 1 New England Biolabs, 240 County Road, Ipswich, MA 01938, USA; 2 Eedetics, 1200 Soldiers Field Road, Boston, MA 02134, USA

## Abstract

**Background:**

Cell-free protein synthesis is not only a rapid and high throughput technology to obtain proteins from their genes, but also provides an *in vitro *platform to study protein translation and folding. A detailed comparison of *in vitro *protein synthesis in different cell-free systems may provide insights to their biological differences and guidelines for their applications.

**Results:**

Protein synthesis was investigated *in vitro *in a reconstituted prokaryotic system, a S30 extract-based system and a eukaryotic system. Compared to the S30 system, protein synthesis in the reconstituted system resulted in a reduced yield, and was more cold-sensitive. Supplementing the reconstituted system with fractions from a size-exclusion separation of the S30 extract significantly increased the yield and activity, to a level close to that of the S30 system. Though protein synthesis in both prokaryotic and eukaryotic systems showed no significant differences for eukaryotic reporter proteins, drastic differences were observed when an artificial fusion protein was synthesized in vitro. The prokaryotic systems failed to synthesize and correctly fold a significant amount of the full-length fusion protein, even when supplemented with the eukaryotic lysate. The active full-length fusion protein was synthesized only in the eukaryotic system.

**Conclusion:**

The reconstituted bacterial system is sufficient but not efficient in protein synthesis. The S30 system by comparison contains additional cellular factors capable of enhancing protein translation and folding. The eukaryotic translation machinery may have evolved from its prokaryotic counterpart in order to translate more complex (difficult-to-translate) templates into active proteins.

## Background

Cell-free protein synthesis has gained increasing popularity as a rapid and high throughput technology to obtain proteins from their genes [1-3]. Cell-free protein synthesis systems often use a cell lysate from *E. coli *cells, rabbit reticulocytes or wheat germ to supply the protein translation machinery and a recombinant T7 RNA polymerase to couple transcription to translation. Perhaps the biggest drawback of synthesizing proteins in the lysate is that the lysate contains a large portion of the cellular proteins and nucleic acids that are not necessarily involved in protein synthesis. How and whether these macromolecules affect the *in vitro *processes are largely unpredictable and often unknown. For instance, proteases and nucleases in the lysates could be inhibitory to protein synthesis. Cellular proteins or nucleic acids in the lysates may interfere with the functional assays and subsequent purification may be hampered by the low amount of the synthesized protein.

As a step closer to addressing these problems, protein translation was reconstituted *in vitro *from purified components of the *E. coli *translation machinery [4]. Except for the ribosomes and tRNAs, which were purified from the *E. coli *lysate, this reconstituted system, appropriately named "the PURE system", contains purified recombinantly-expressed proteins of all *E. coli *translation factors and aminoacyl-tRNA synthetases [4]. Remarkably, this reconstituted system has been shown to catalyze efficient *in vitro *protein synthesis [5]. Largely free of other cellular components, the PURE system facilitates *in vitro *studies in a much cleaner background than a lysate-based system. The immediate impact of the PURE system and other similar reconstituted systems was their superior performance in such *in vitro *applications as the incorporation of unnatural amino acids [6], ribosome display [7,8] and mRNA or pure translation display [9,10], largely due to their designability and their significantly reduced nuclease and protease activities.

In spite of the advantages of the PURE system, we suspected that this "stripped-down" version of the protein translation machinery would encounter problems when "difficult" templates for translation were used. In this study, we compared protein synthesis of several proteins that exhibited significant differences when synthesized in the PURE system and an S30 system. An S30 system is an *E. coli *extract-based system, derived from the *E. coli *cell lysate obtained after 30,000 × g centrifugation [11]. To begin to investigate the reasons for these differences, we added the size-separated fractions of an *E. coli *lysate to the *in vitro *reactions of the PURE system. Our data suggest the possibility of additional factors that further promote *in vitro *protein synthesis.

Many eukaryotic proteins that fold correctly in eukaryotes tend to misfold when expressed in *E. coli*. Such capability of eukaryotes, which has evolved to accommodate the needs for more complexity in proteins, has been attributed, at least in part, to the cooperativity of a large number of eukaryotic chaperones and their intimate association with translation and eukaryotic ribosomes [12]. To investigate the differences in protein synthesis between prokaryotic and eukaryotic *in vitro *systems, we synthesized a fusion protein consisting of two eukaryotic protein domains, which was found to fold well in a rabbit reticulocyte system, but not in an *E. coli *system. In particular, we asked if this fusion protein translated by *E. coli *ribosomes would fold better in the presence of eukaryotic chaperones. Such experiments would be difficult to perform in vivo, as successful co-expression of a complete set of eukaryotic chaperones in *E. coli *is perhaps an impossible task by itself. We instead performed *in vitro *protein synthesis in the S30 system to which a eukaryotic lysate or its fractions were added. Our data illustrated striking differences in protein synthesis between prokaryotic and eukaryotic *in vitro *systems.

## Results and discussion

### Compared to the S30 system, the prokaryotic reconstituted system (the PURE system) produced noticeably less protein, and was more cold-sensitive

**Figure 1:**
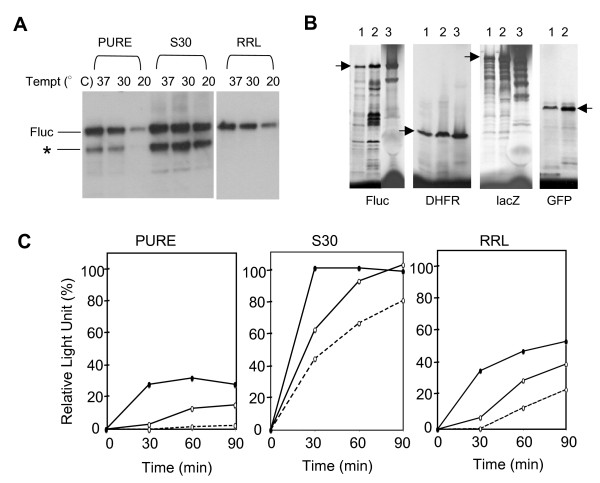
***In vitro *synthesis of target proteins in different cell-free systems**. A. Western blot analysis of *in vitro *synthesis of Fluc at 60 min. at three temperatures in the PURE system (PURE), the S30 and RRL systems. B. Different reporter proteins (indicated) were synthesized for 60 min. in the reactions containing ^35^S-methionine in the PURE system (lane 1), the PURE system supplemented with the fraction 17 (lane 2) and in the S30 system (lane 3). Aliquots of the reactions were run on a SDS-PAGE gel, followed by autoradiography. The arrows indicate the positions of the full-length proteins. C. A time course of *in vitro *synthesis of Fluc in different systems (indicated) and at three temperatures (37°C, filled ovals with solid line; 30°C, open ovals with solid line; 20°C, open ovals with dash line). The activities are averages of duplicate experiments and presented as the percentage of the relative light unit determined at 37°C and 60 min.

Firefly luciferase (Fluc), a 61 kDa two-domain eukaryotic protein [13], was chosen as a model protein for *in vitro *synthesis. The same circular plasmid containing a T7 promoter, a Shine-Dalgarno (SD) sequence before the ATG start site and the gene for Fluc, was used as the DNA template for *in vitro *protein synthesis in the PURE system, the S30 system (S30) and the rabbit reticulocyte lysate system (RRL) (Fig. 1). The full-length active protein was synthesized in all three systems as judged by the western blot and the activity assay (Fig. 1A &1C). A truncated Fluc product (indicated by an asterisk * in Fig. 1A), detected by the western blot in the PURE system and S30 but not in RRL, was probably a product of the translation at an internal translation start site. Consistent with this notion, fusion of a small protein domain to the C-terminus but not the N-terminus of Fluc shifted the truncated protein band to a higher molecular weight (data not shown). The truncated Fluc product was not detected in RRL (Fig. 1A), suggesting the putative internal start site was not recognized by the eukaryotic ribosomes. The lower yield in RRL compared to that of S30 was probably due to the fact that no Kozak sequence was present in the translation initiation site of the Fluc gene [14].

Significantly less protein and lower activity were observed in the PURE system than in S30 for Fluc and some other proteins (Fig. 1A &1C, B lane 1 and 3). The PURE system contains the minimal set of components necessary for *in vitro *protein synthesis, lacking numerous other factors that are involved in protein synthesis and nascent chain folding in vivo. A previous study showed that the major *E. coli *chaperones were largely absent in the PURE system, and addition of DnaK family chaperones and trigger factor to the PURE system enhanced the activity of a synthesized protein [15]. It is possible that the presence of chaperones in S30 only accounted for the enhanced activity of already synthesized proteins, whereas the increase in the overall protein synthesis yield was due to other factors in S30.

Another interesting difference between the PURE system and S30 was that protein synthesis in S30 was less affected by low temperatures than in the PURE system. During the first 30 min. of protein synthesis, the difference in the initial accumulation of the activity (and the yield (not shown)) between 20°C and 37°C was much smaller in S30 than in the PURE system (Fig. 1C). At 20°C, almost no activity and protein (not shown) were observed during the first 30 min. of the synthesis in the PURE system (Fig. 1C). Even after 1 hr at 20°C, only a small amount of Fluc was detected in the PURE system (Fig. 1A). It is possible that this cold-sensitivity of the PURE system was due to its lack of certain cellular factors whose in vivo functions are to maintain efficient translation after cold shock [16].

### Supplementing the PURE system with the *E. coli *cell extract or its fractions improved the yield and activity to a level close to that of the S30 system

**Figure 2:**
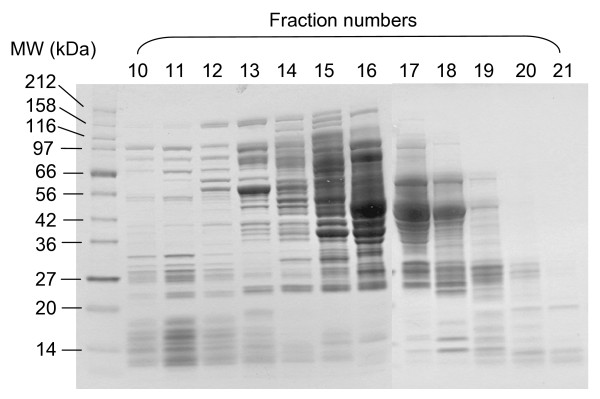
**SDS-PAGE analysis of the peak fractions (10 to 21) from the size-exclusion separation of an S30 extract**. The molecular weight standards for the SDS-PAGE are indicated on the left. Based on the molecular weight standards for the size exclusion column, fractions 9–11 are >700 kDa; fractions 12–13 are 600–200 kDa; fractions 14–20 are 200–10 kDa.

**Figure 3:**
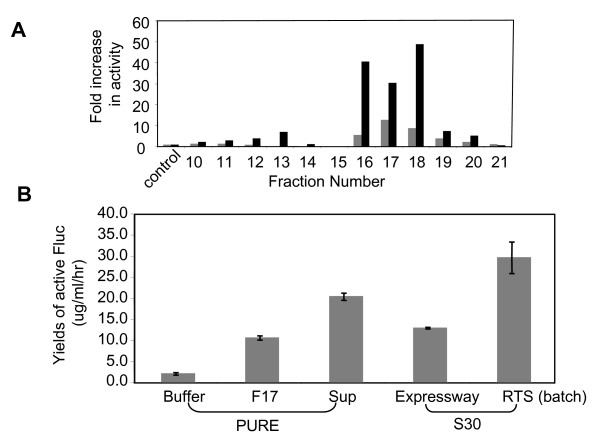
**The effect of the size-separated fractions on *in vitro *protein synthesis**. A. Screening the fractions (10 to 21) from a size-exclusion separation of an S30 extract for enhancement in *in vitro *protein synthesis in the PURE system. The activities were assayed at 60 min. for Fluc (grey bars) and β-galactosidase (black bars), and by comparing to the activities of the controls (supplemented with only the column buffer), the data are presented as "fold increase". B. Comparison of the protein synthesis yields of Fluc between the PURE system (PURE) without any supplement (Buffer) or supplemented with the fraction 17 (F17) or the S30 extract (Sup), with the S30 systems from Invitrogen (Expressway) and Roche (RTS). The yields were calculated from the activities of the *in vitro *synthesis reactions compared to that of the known amount of pure Fluc protein assayed at the same time.

A S30 extract (Sup), generated from growing *E. coli *cells, was fractionated on a size-exclusion column. Each of the peak fractions (fractions 10 to 21) (Fig. 2) was concentrated and then added (10% v/v) to the *in vitro *synthesis reactions of the PURE system. The activities were determined for *E. coli *β-galactosidase (lacZ) and Fluc. As shown in Fig. 3A, supplementing fractions 16, 17 and 18 resulted in a significant enhancement in the activities of both proteins. The fractions themselves had no detectable luciferase and galactosidase activity (not shown). The fraction 17 was also shown to enhance the yields of Fluc and several other proteins (Fig. 1B, lane 2).

The effects of the fraction 17 (F17) and the extract (Sup) on the amount of synthesized Fluc in the PURE system were compared with two other commercial S30 systems (Fig. 3B). The addition of the fraction (F17) or the extract (Sup) significantly increased the *in vitro *synthesis yields in the PURE system to a level close to those of the S30 systems (Fig. 3B).

The SDS-PAGE gel analysis of the peak fractions suggested that the fractions 16 to 18 contained the bulk of the *E. coli *soluble proteins, consistent with their size range of ~10–150 kDa (Fig. 2). The data indicated that the "active ingredients" were likely to be macromolecules such as proteins, but not the small molecules, which were eluted at later fractions (data not shown). These "active" macromolecules could include those already present in the PURE system, such as translation factors, chaperones, and those lacking in the PURE system, such as other cellular factors that are important for efficient protein synthesis. Consistent with this notion, supplementing the PURE system reactions with F17 or Sup also significantly enhanced the protein synthesis yields at 20°C (data not shown). Since the PURE system is a coupled transcription/translation system, it is also possible that *in vitro *transcription was affected in such a way that protein translation was enhanced [17].

### Supplementing the S30 system with the rabbit reticulocyte lysate or its fractions failed to produce an active full-length fusion protein

**Figure 4:**
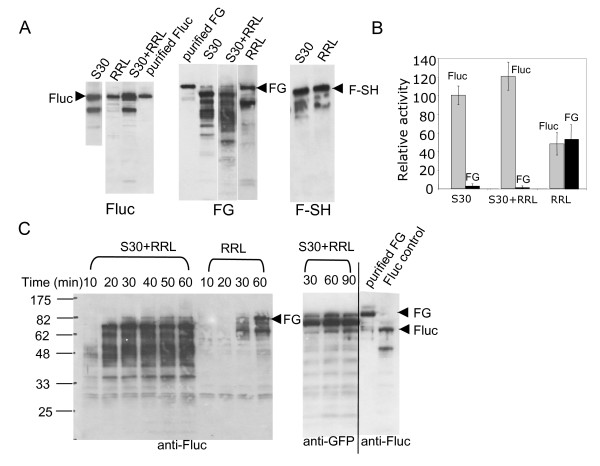
***In vitro *synthesis of the fusion protein FG**. A. Western blot analyses (anti-Fluc) of the *in vitro *synthesis reactions (at 60 min.) of Fluc, FG and F-SH in different systems. The full-length proteins, indicated by arrows, were confirmed by using the purified proteins of Fluc (purified Fluc) and FG (purified FG). B. The Fluc activities were determined for the Fluc and FG templates after 60 min. of the *in vitro *synthesis reactions in different systems. The data are presented as the relative activities with the activity of Fluc in S30 at 60 min being 100. C. Western blot analyses of the time courses of the *in vitro *synthesis reactions of FG in S30+RRL and RRL using anti-Fluc and anti-GFP to probe the translation products. The positions of the full-length FG and Fluc, indicated by arrows, were confirmed by the purified FG (purified FG) and the *in vitro *synthesis reaction of Fluc (FLuc control). The molecular weights (kDa) are indicated at the left.

There was no significant difference in the specific activity (the activity normalized by the yield) when Fluc was synthesized in either S30 or RRL at 37°C (Fig. 1A and 1C). However, when a fusion protein (FG), consisting of the Fluc and GFP domains, was synthesized, drastic differences were observed (Fig. 4). No significant amount of the full-length fusion protein and almost no activity were observed in S30, whereas the full-length fusion protein and the Fluc activity were evident in RRL (Fig. 4A &4B). It seemed that the Fluc domain in FG was correctly folded when synthesized in RRL, but not in S30. This appeared to be due to the GFP domain, since another fusion protein (F-SH), which had a similar size as FG and consisted of the Fluc and the SH2–SH3 domains, was successfully synthesized in both S30 and RRL (Fig. 4A, right panel).

To investigate if the ability of RRL to synthesize the full-length FG was due to its translation or its folding machineries, S30 was supplemented with a crude rabbit reticulocyte lysate, resulting in a mixed system (S30+RRL). Cycloheximide, an antibiotic specific to the eukaryotic translation, was added to S30+RRL to prevent any residual translation from the eukaryotic ribosomes. S30+RRL was capable of synthesizing the full-length and active Fluc (Fig. 4A &4B). It appeared that in S30+RRL, protein translation occurred on the bacterial ribosomes, as the truncated Fluc product, seen only in S30, was also observed in S30+RRL (Fig. 4A, left panel). In addition, the protein synthesis in S30+RRL was sensitive to the inhibition by chloramphenicol, an antibiotic specific to prokaryotes (data not shown). It was apparent that the prokaryotic translation machinery was fully functional in S30+RRL, and under such conditions, the nascent chains from the *E. coli *ribosomes could be the substrates for folding by the eukaryotic chaperones present in the rabbit reticulocyte lysate supplement. However, when the fusion protein FG was synthesized in S30+RRL, no significant amount of the full-length FG and almost no Fluc activity were detected (Fig. 4A, middle panel, and 4B). This result remained the same whether the crude RRL (Pel-Freez) or the RRL from the in vitro protein synthesis kit (Promega), which presumably contained functional eukaryotic chaperones, was used as the supplement (data not shown). Lowering the temperatures (to 30°C or 23°C) to slow down the rate of translation from the *E. coli *ribosomes failed to make any difference in the case of FG in both S30 and S30+RRL (data not shown). In fact, the elongation rate of translation in S30 has been estimated to be similar to that of eukaryotic translation [18].

**Figure 5:**
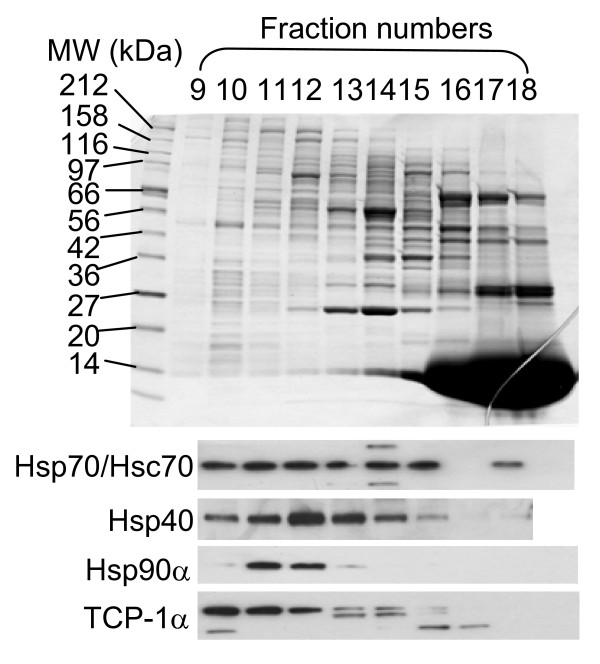
**Analysis of rabbit reticulocyte lysate by size separation and western blotting**. Top, SDS-PAGE analysis of the peak fractions (9 to 18) from the size-exclusion separation of a crude rabbit reticulocyte lysate. The molecular weight standards for the SDS-PAGE are indicated on the left. Based on the molecular weight standards for the size exclusion column, fractions 9–11 are >700 kDa; fractions 12–13 are 600–200 kDa; fractions 14–20 are 200–10 kDa. Bottom, western blot analyses of these fractions (9–18) using antibodies against Hsp70/Hsc70, Hsp40, Hsp90a and TCP-1a.

Previous studies suggest that in eukaryotes sequential and co-translational folding of a fusion protein allows correct folding of its constituent domains, whereas in prokaryotes, the post-translational folding of the same fusion protein results in intramolecular misfolding due to concurrent domain folding [19]. The reason may be that eukaryotic chaperones are uniquely recruited to the translating ribosomes to ensure that translation and nascent chain folding are coordinated [12,20]. Consistent with these studies, we detected several known eukaryotic chaperones by western blot in the fractions of RRL after a size-exclusion chromatography (Fig. 5). It was surprising that these chaperones were mostly not in the fractions that corresponded to their molecular weights, but in those of much larger molecular weights and in those containing ribosomes (Fig. 5) [21]. None of these fractions when supplemented in S30 had a significant effect on the synthesis of Fluc or FG (data not shown). It is possible that eukaryotic chaperones are effective in nascent chain folding only when protein translation occurs on eukaryotic ribosomes.

Western blot analyses of the *in vitro *synthesis reactions of FG in S30 or S30+RRL using anti-Fluc revealed a large number of products corresponded to a wide range of molecular weights shorter than the full length FG (Fig. 4C). These incomplete Fluc products were largely not seen in RRL (Fig. 4C). When anti-GFP was used to probe these products, only those with molecular weights larger than Fluc were detected, whereas those smaller than Fluc were not (Fig. 4C). These data suggest that these smaller truncated translation products were likely the result of premature termination or pausing during translation, not the degradation products of the synthesized full-length fusion proteins. Similar results were also observed by other studies [22].

Why did a large number of the elongating ribosomes in S30 or S30+RRL stop on the template of FG even before the full-length Fluc was synthesized (Fig. 4C), even though most ribosomes were capable of completing the synthesis on the templates of Fluc (Fig. 4A, left panel) and a fusion protein F-SH (Fig. 4A, right panel)? Since both the Fluc and GFP genes can be readily expressed on their own in S30 (Fig 1B), we speculate one possibility is that the sequence of GFP when fused to that of Fluc created a complex template, *e.g.*, potential inhibitory structures on the mRNA template, or uniquely misfolded nascent chain, causing the *E. coli *ribosomes to pause or terminate prematurely, often even before the upstream Fluc domain was translated. The presence of eukaryotic chaperones or other factors has no effect as long as the translation was from the *E. coli *ribosomes (Fig. 4C). The eukaryotic ribosomes, perhaps with their associated chaperones and other factors, seemed to have the ability to overcome these obstacles.

## Conclusion

*In vitro *protein synthesis was compared not only between the minimal PURE system and the extract-based system, but also between the prokaryotic and eukaryotic systems. The advantage of the *in vitro *approach allowed us to supplement one system with the lysates or their fractions from another system, thereby providing insights to the fundamental differences between these systems. Since ribosomes, translation factors and aminoacyl-tRNA synthetases are among the most conserved RNA and protein molecules, the PURE system may resemble an ancestral form of the minimal protein translation machinery, which, as this study suggests, is sufficient but not efficient in protein synthesis. The S30 system by comparison contains the modern bacterial version, with additional cellular factors capable of enhancing protein translation and folding. The eukaryotic translation machinery may have evolved from its prokaryotic counterpart in order to translate more complex (difficult-to-translate) templates into active proteins.

## Methods

### Materials

The reagents for making the constructs of Fluc and the fusion proteins were from New England Biolabs. All antibodies against chaperones were purchased from StressGen Bioreagents (Ann Arbor, MI). Monoclonal anti-Fluc and anti-GFP antibodies, cycloheximide, and chloramphenicol were purchased from Sigma. The S30 system and the RRL system were purchased from Promega except when indicated otherwise. Other S30 systems used were Expressway (Invitrogen) and the RTS system (Roche biosciences). A crude rabbit reticulocyte lysate was purchased from Pel-Freez Biologicals (Rogers, AR). The PURE system (Classic II) was purchased from Post Genome Institute (Tokyo, Japan). ^35^S-methionine (37 Tbq/mmol) was from Amersham.

### Plasmid constructs of the reporter proteins

The genes for firefly luciferase (61 kDa) (Fluc), *Aequorea victoria *GFP (27 kDa), and *E. coli *β-galactosidase (116 kDa) (lacZ) were cloned into a pUC19-derived vector containing a T7 promoter and a bacterial Shine-Dalgarno sequence. The vector for *in vitro *synthesis of *E. coli *dihydrofolate reductase (DHFR) was a control vector in the PURE system kit. The fusion protein FG (88 kDa) was constructed by fusing the N-terminus of GFP to the C-terminus of Fluc. Another fusion protein F-SH (81 kDa) consisted of the SH2–SH3 domain of human p120 (GAP) protein fused to the C-terminus of Fluc.

### *In vitro *protein synthesis reactions

All reactions were conducted following the recommended protocols by the manufacturers of the *in vitro *systems. All S30 systems were used in batch mode. 400–500 ng of the plasmid DNA was used for each 50 μl reaction. For ^35^S labeling, 4 μl ^35^S-methionine was added per 50 μl reaction. For the PURE system reactions containing the supplements, 2.5 μl of the S30 extract (Sup) or the concentrated fraction was used for each 25 μl reaction. The S30 extract was prepared from an exponentially growing *E. coli *strain according to the protocol of Zubay [11] and obtained from the laboratory of Chris Noren (New England Biolabs). For *in vitro *reactions in a mixed system (S30+RRL), 10 μl of the rabbit reticulocyte lysate from Pel-Freez or Promega and 2 ng/μl cycloheximide was added to the S30 reaction mixture in a final volume of 50 μl.

### Activity assays

The Fluc activity was assayed using the Luciferase Assay System (Promega) and a microplate luminometer (Centro LB 640, Berthold Technologies). The same luminometer was also used to assay the lacZ activity in combination with the Galacto-Light™ system (Applied Biosystems).

### Analysis of the synthesized proteins

Aliquots (1 μl for the western blot analyses or 5 μl for ^35^S labeling) were taken from the *in vitro *reactions and loaded on 10–20% Tris-glycine SDS PAGE gels (Invitrogen). The gels were either blotted for analyses with antibodies or dried for exposure to X-ray films.

### Size-exclusion chromatography

For fractionation of the S30 extract, 1 ml of the extract (~12 mg/ml protein) in the column buffer (20 mM HEPES, pH 7.6, 50 mM KCl, 1 mM DTT) was loaded onto a Superdex 200 (10/300) column (Pharmacia). The fractions were collected and then concentrated 5–10 fold in Centricon tubes (Milipore). For fractionation of the crude rabbit reticulocyte lysate, 1 ml of the lysate in the RRL column buffer (30 mM HEPES, pH 7.2, 10 mM NaCl, 1 mM MgCl_2_, 0.1 mM EDTA, 25 mM KOAc, and 0.2 mM DTT) was loaded on the same column. The fractions were directly used for western blot analyses.

## Abbreviations

S30: an *E. coli *extract-based *in vitro *protein synthesis system; RRL: rabbit reticulocyte lysate or rabbit reticulocyte lysate-based *in vitro *protein synthesis system; S30+RRL: S30 supplemented with RRL; The PURE system: a reconstituted *in vitro *protein synthesis system based on protein translation in *E. coli*; Fluc: firefly luciferase; GFP: green fluorescent protein;

## Authors' contributions

JRH performed most of the experiments. SC performed some experiments using the PURE system. SC wrote the manuscript.
